# Immunohistochemical expression of MMP-2 and MMP-8 in oral squamous cell carcinoma

**DOI:** 10.4317/jced.52047

**Published:** 2015-04-01

**Authors:** Ahmed-Oluwatoyin Lawal, Akinyele-Olumuyiwa Adisa, Bamidele Kolude, Bukola-Folasade Adeyemi

**Affiliations:** 1 (FMCDS), Lecturer/Consultant, Department of Oral Pathology, College of Medicine, University of Ibadan, Nigeria; 2 (FWASC), Lecturer/Consultant, Department of Oral Pathology, College of Medicine, University of Ibadan, Nigeria

## Abstract

**Background:**

Matrix metalloproteinases (MMPs) are endopeptidases that can degrade extracellular matrix components and affect invasiveness and aggressiveness of oral squamous cell carcinoma (OSCC). The aim of this study was to examine the immunohistochemical expression of MMP-2 and MMP-8 in OSCCs in patients presenting at the Tertiary Health facility in Nigeria.

**Material and Methods:**

Formalin-fixed, paraffin-embedded (FFPE) OSCC samples diagnosed between the years 2010 and 2012 were used for his study. The FFPE were processed for MMP-2 and MMP-8 using the specifications of the manufacturer. Two investigators reviewed the slides scoring the pattern and intensity of staining as negative (0), weakly positive (+1), moderately positive (+2) and strongly positive (+3). The data were analysed using version 20 of the SPSS. The level of significance was set at P < 0.05.

**Results:**

Twenty-five OSCC consisting of 14 (56%) males and 11 females (44%) were used. The mean age was 54.6 ± 17.9 years. A higher proportion (100%) of poorly differentiated OSCC strongly expressed MMP-2 compared with the well differentiated and moderately differentiated OSSC. There was no significant difference in the expression of MMP-2 amongst the three grades of OSCC (X2 = 2.87; p= 0.17). Only 5 (20%) OSCC cases positively expressed MMP-8. Moderate expression of MMP-8 was only seen in well-differentiated OSCCs.

**Conclusions:**

This study showed that a higher proportion of poorly differentiated OSSC strongly expressed MMP-2. Eighty percent of cases that express MMP-8 were females and moderate expression of MMP-8 was seen only in well differentiated OSCC.

** Key words:**Oral squamous cell carcinoma, MMP-2, MMP-8, immunohistochemistry.

## Introduction

Oral squamous cell carcinoma (OSCC) is the most common malignant neoplasm in the oral cavity, and it is still regarded as a significant global public health problem (1). The management of OSCC most often includes surgery with or without neck dissection, followed by adjuvant radiotherapy (2). In spite of diagnostic and therapeutic developments, the 5-year survival rate for head and neck cancers has not significantly improved over the past decades (2,3).

Matrix metalloproteinases (MMPs) are a family of at least 17 human zinc dependent endopeptidases that can degrade almost all extracellular matrix components (4). They are classified into five groups according to their substrate specificity: collagenases (MMP-1, -8, and -13), gelatinases (MMP-2 and -9), stromelysins (MMP-3, -10), and membrane-type MMPs (MT-MMPs) (MMPs 14–17, 24, and 25) and other MMPs (MMPs11, 19 and 20) (5). The expression of these MMPs is rapidly induced when active tissue remodelling is required in physiological and pathological conditions (5,6). It is well recognized that MMPs are key mediators of tumour invasion and metastasis because of their involvement in cell proliferation, survival, angiogenesis, and cell migration (6).

Fan et al. (7) in a study in China had previously reported that high tumour and stromal MMP-2 and MMP-9 expression were significantly associated with positive lymph node status and the overall patient survival was significantly shorter in patients with high tumour and stromal MMP-2 and MMP-9 expression. Furthermore, MMPs inhibitors are potential therapeutic agents in the management of cancers with less adverse effects and possible better prognosis. This present study was carried out to examine the immunohistochemical expression of MMP-2 and MMP-8 in oral squamous cell carcinoma cases seen at the Department of Oral Pathology, University College Hospital Ibadan.

## Material and Methods

Twenty-five samples of formalin-fixed, paraffin-embedded (FFPE) OSCC diagnosed between the years 2010 and 2012 were retrieved from the Department of Oral Pathology archives, University College Hospital, Ibadan. Freshly prepared sections were stained with haematoxylin–eosin (H&E) and the antibody to MMP-2 (Monoclonal CA-4001/CA719E3C, Abcam), MMP-8 (Monoclonal 115-13D2, Abcam) using the specifications of the manufacturer. The sections for immunohistochemistry were deparaffinized, hydrated and then rinsed in phosphate-buffered solution (PBS). They were immersed in heat-induced epitope retrieval citrate buffer diluted 1:10 with distilled water and incubated at 900 C for 60 minutes. They were then placed in fresh citrate, cooled in water for 20 minutes and then rinsed in PBS. Positive and negative controls were employed for the antibodies.

Hydrogen peroxide (3%) was added to each section for 10 minutes, and the sections were rinsed in 0.1% PBS. The specimens were incubated for 60 minutes with 1:20 dilution of Abcam mouse monoclonal antibody to MMP-2 and MMP-8 followed by incubation with undiluted labelled polymer horseradish peroxidase-conjugated with anti-mouse secondary antibody for 30 min. One ml of diaminobenzidine solution was added to cover the specimen, followed by incubation in a humidity chamber for 15 minutes. The sections were then immersed in aqueous haematoxylin and rinsed in distilled water. The tissue was dehydrated and subsequently rinsed with xylene. Distyrene plasticizer in xylene mounting fluid was then applied, and a cover slip placed.

Two investigators reviewed the slides scoring the pattern and intensity of staining as follows: negative (<10%) (0), weakly positive (10–25%) (+1), moderately positive (25–50%) (+2) and strongly positive (>50%) (+3) (8). The data were analysed using version 20 of the SPSS. Qualitative data were compared using chi-squared statistics. Quantitative data were summarized using mean, standard deviation and confidence interval and compared using Mann-Whitney U and/or one-way analysis of variance test. The level of significance was set at *P* < 0.05.

## Results

A total of 25 cases of OSCC consisting of 14 (56%) males and 11 females (44%) were included in this study. The age range was 10-86 years with a mean age of 54.6 ± 17.9 years. The palate was the most common site of occurrence with 6 (24%) cases followed by the mandible with 5 (20%) cases while the maxilla and lip mucosa both had 4 (16%) cases each and other sites such as tongue, buccal mucosa, floor of mouth, maxillary antrum altogether accounted for 7 (20%) cases. The moderately differentiated OSCC with 13 (52%) cases was the most common grade followed by the well differentiated with 10 (40%) cases, while the poorly differentiated OSCC accounted for 8% of cases ( Table 1).

**Table 1:**
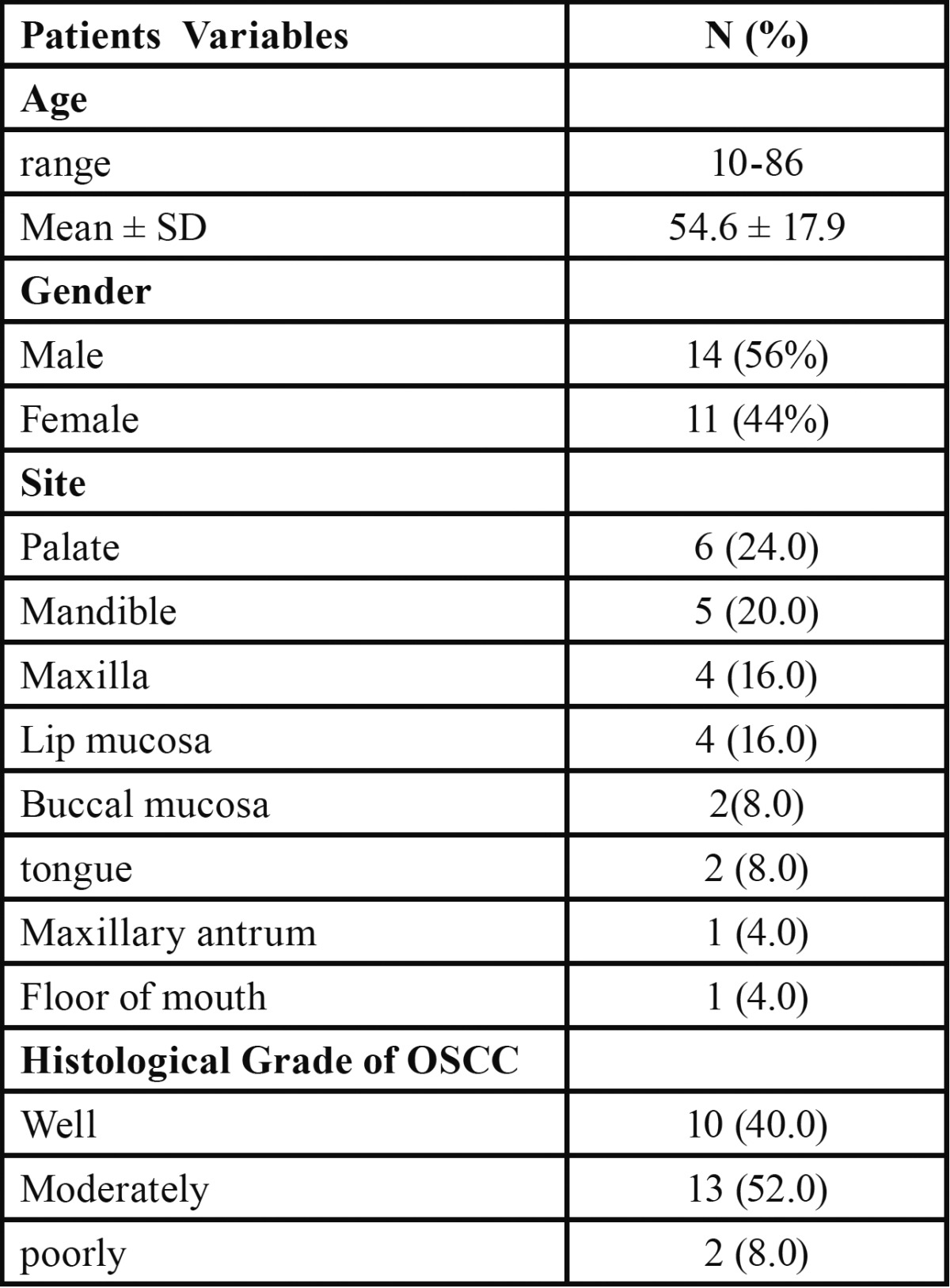
Demographic and clinicopathological characteristics of patients.

 Table 2 shows the expression of MMP-2 in different grades of OSCC. Eight (80%) well-differentiated OSCC showed positive expression for MMP-2, 61.5% of moderately differentiated OSSC showed positive expression while 100% of poorly differentiated OSCC showed positive expression for MMP-2. The two cases (100%) of poorly differentiated OSCC strongly expressed MMP-2 while only 40% and 23.1% of the well differentiated and moderately differentiated OSSC strongly expressed MMP-2 respectively. There was however, no significant difference in the expression of MMP-2 amongst the three grades of OSCC (X2 = 2.87; *p*= 0.17). Also, there was no statistically significant difference in MMP-2 expression according to gender and site (*p*=0.23 and *p* = 0.37 respectively Mann-Whitney U).

**Table 2:**
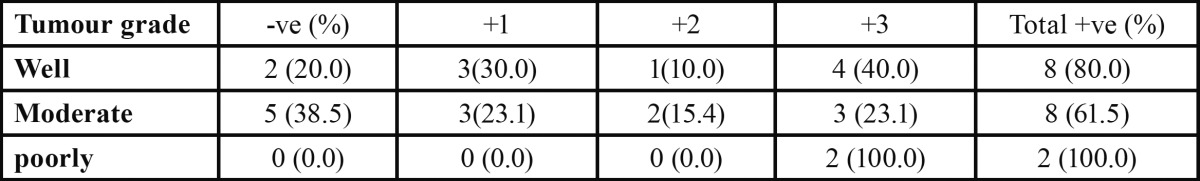
Expression of MMP-2 according to histologic grade.

**Figure 1:**
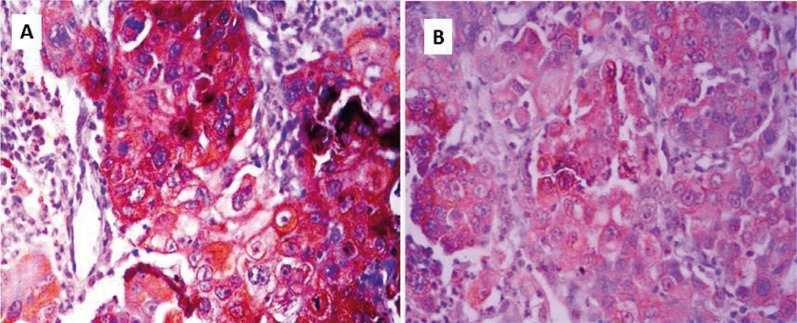
Immunohistological findings for MMP2, MMP8 in oral squamous cell carcinoma. (A and B) Immunoperoxidase stain with hematoxylin counterstain in well differentiated squamous cell carcinoma with (+++) MMP-2 stain and moderately differentiated squamous cell carcinoma with (++) MMP-8 stain.

Only 5 (20%) OSCC cases positively expressed MMP-8 while 20 (80%) were negative. Three (30%) well-differentiated OSCC showed positive expression for MMP-8, one of the 13 (7.7%) cases of moderately differentiated OSSC showed positive expression while one of the two cases (50%) of poorly differentiated OSCC showed positive expression for MMP-8. Of the 5 cases that were positive for MMP-8, 3 (12%) mildly expressed MMP-8, 2 (8%) moderately expressed MMP-8 while no case strongly expressed MMP-8. The two cases that moderately expressed MMP-8 were well-differentiated OSCCs. All the three cases of OSCC that mildly expressed MMP-8 strongly expressed MMP-2. Although, majority (80%) of the cases that expressed MMP-8 were seen in females there was no statistically significant difference in the expression of MMP-8 between males and female (*p*=0.065 Mann-Whitney U). Figure 1 shows MMP-2 and MMP-8 expressions in well differentiated OSCC and moderately differentiated OSCC respectively.

## Discussion

The palate was commonest site of occurrence for OSCC in this study which was contrary to many previous reports that showed the tongue to be the most common site of occurrence (9-11). However, studies from Africa (12,13) have shown other sites aside from the tongue to be the most common sites of occurrence and a study by Otoh *et al.* (14) from Nigeria which reported that the palate was commonest site was in agreement with this study. Although, the finding of a mean age of 54.6 years was in conformity with the fact that OSCC is more commonly seen in older patients especially in those above age of forty (10,12), the age range of 10-86 shows it can also be found in the very young. Previous reports have also shown that OSCC can occur in very young with some authors reporting OSCC in children as young as 3 years of age (12,13). It is known that an inherited bone marrow failure syndrome, dyskeratosis cogenita may predispose sufferers to squamous cell carcinoma which usually present between the ages 5-12 years (15). However the presence of dyskeratosis cogenita or any other congenital predisposing factor was not proven in this case.

There was a relatively higher proportion of strong expression of MMP-2 in poorly differentiated OSCC in this study compared to the other histologic grades. A study in Iran reported a positive correlation between levels of MMP-2 expression and histologic grades of OSCC (16). Similarly, in a study considering expression of p53, Ki-67, MMP-2 and MMP-9 in OSCC and verrucous carcinoma, it was reported that high grade OSCCs had the highest overall mean of expression compared to low-grade OSCCs and verrucous carcinomas (17). The high expression of MMP-2 by tumour cells translates to greater degradation of basement membrane (BM) and extracellular matrix (ECM), thus providing channels that allow tumour cells to migrate and metastasize by direct spread and in vascular systems (18).

In contrast, a study from China (19), found that MMP-2 expression was greater in the less aggressive verrucous carcinomas when compared with OSCC. In addition, they found no differences in MMP-2 expression among poor, moderately and well-differentiated OSCC. These contradictory findings may be due to differences in techniques of tissue fixation and antigen retrieval. It is also possible that racial and genetic differences among varying sub-populations may influence expression of MMP-2 in different grades of OSCC.

MMP-8, unlike most other MMPs, has been shown to exhibit antitumor properties (20). Montel *et al.* (21) described tumour spread inhibition by MMP-8 in animal breast cancer models. A high plasma level of MMP-8 has been shown to be protective against lymph node metastasis in breast cancer patients (8). A study reported that an elevated MMP-8 expression in tongue carcinoma cells was associated with an increased survival (22). On the contrary, Pradhan-Palikhe *et al.* (20) found that plasma MMP-8 levels did not predict survival in head and neck squamous cell carcinoma. Although the anti-tumour and anti-invasive mechanisms of MMP-8 is not known with certainty, Korpi *et al.* (22) suggested that the protective role of MMP-8 may be related to its regulation of estrogen receptor (ER) signalling, especially in hormone-regulated malignancies. Thus they suggested that its tumour inhibiting properties might be more pronounced in females. Although, we found no statistically significant difference in expression of MMP-8 between males and females, majority of the cases that expressed MMP-8 were seen in females. We also observed that cases that mildly expressed MMP-8 also strongly expressed MMP-2 and this suggests a counteractive effect of MMP-2 and MMP-8 on each other.

The use of MMP inhibitors (MMPIs) in cancer therapy, though produced some encouraging results in vitro, were disappointing in clinical trials (23,24). These failures have been attributed to the fact that some of these MMPIs may confer long-term side effects by disrupting some host-protective properties (23). Also, the fact that some MMPs (such as MMP-8) produced some anti-tumour properties was not put into consideration in some of these previous treatments. However, the advent of selective MMPIs (24-26) may help in resolving some of these downsides. It has also been suggested that the model of using biologic agents with molecularly targeted activity in selected cancer cases as in the use of trastuzumab (Herceptin) in HER-2/Neu positive breast cancer could also improve effectiveness of these MMPIs (26).

This study showed that a higher proportion of poorly differentiated OSSC strongly expressed MMP-2 and strong expression of MMP-2 may correlate with aggression of OSCC. On the other hand, most cases did not express MMP-8 and moderate expression of MMP-8 was seen only in well differentiated OSCC. More so, MMP-8 was expressed predominantly in females and eighty percent of cases that express MMP-8 were females. We suggest that using selective MMP-2 inhibitors in OSCC that strongly express MMP-2 may achieve better results than obtained from previous clinical trials of MMPIs in cancer therapy.
